# Pegylated Insulin-Like Growth Factor 1 attenuates Hair Cell Loss and promotes Presynaptic Maintenance of Medial Olivocochlear Cholinergic Fibers in the Cochlea of the Progressive Motor Neuropathy Mouse

**DOI:** 10.3389/fneur.2022.885026

**Published:** 2022-06-03

**Authors:** Linda Bieniussa, Baran Kahraman, Johannes Skornicka, Annemarie Schulte, Johannes Voelker, Sibylle Jablonka, Rudolf Hagen, Kristen Rak

**Affiliations:** ^1^Department of Oto-Rhino-Laryngology, Plastic, Aesthetic and Reconstructive Head and Neck Surgery, University Hospital Würzburg, Würzburg, Germany; ^2^Department of Neurology, University Hospital Würzburg, Würzburg, Germany; ^3^Institute of Clinical Neurobiology, University of Würzburg, Würzburg, Germany

**Keywords:** cochlea, microtubules, MOC fibers, hearing loss, pegylated insulin-like growth factor 1, outer hair cell (OHC), motor neuropathy

## Abstract

The progressive motor neuropathy (PMN) mouse is a model of an inherited motor neuropathy disease with progressive neurodegeneration. Axon degeneration associates with homozygous mutations of the TBCE gene encoding the tubulin chaperone E protein. TBCE is responsible for the correct dimerization of alpha and beta-tubulin. Strikingly, the PMN mouse also develops a progressive hearing loss after normal hearing onset, characterized by degeneration of the auditory nerve and outer hair cell (OHC) loss. However, the development of this neuronal and cochlear pathology is not fully understood yet. Previous studies with pegylated insulin-like growth factor 1 (peg-IGF-1) treatment in this mouse model have been shown to expand lifespan, weight, muscle strength, and motor coordination. Accordingly, peg-IGF-1 was evaluated for an otoprotective effect. We investigated the effect of peg-IGF-1 on the auditory system by treatment starting at postnatal day 15 (p15). Histological analysis revealed positive effects on OHC synapses of medial olivocochlear (MOC) neuronal fibers and a short-term attenuation of OHC loss. Peg-IGF-1 was able to conditionally restore the disorganization of OHC synapses and maintain the provision of cholinergic acetyltransferase in presynapses. To assess auditory function, frequency-specific auditory brainstem responses and distortion product otoacoustic emissions were recorded in animals on p21 and p28. However, despite the positive effect on MOC fibers and OHC, no restoration of hearing could be achieved. The present work demonstrates that the synaptic pathology of efferent MOC fibers in PMN mice represents a particular form of “efferent auditory neuropathy.” Peg-IGF-1 showed an otoprotective effect by preventing the degeneration of OHCs and efferent synapses. However, enhanced efforts are needed to optimize the treatment to obtain detectable improvements in hearing performances.

## Introduction

Sensorineural hearing loss is the most frequent sensory disease in humans. So far, no causative therapies exist. Prospective therapeutic options for auditory dysfunction need to be based on profound knowledge about the organization and function of the cochlea. For this purpose, animal models with genetic hearing defects play an important role (1). The homozygous progressive motor neuropathy (PMN) mouse, which has been originally described as a model for a fast-developing motoneuron disorder the non-5q- spinal muscular atrophy (SMA) (2, 3), represents such a model. The motoneuron disease of non-5q-SMA patients is caused by a spontaneous point mutation (c.1682T>G) in the tubulin-specific chaperone E (TBCE) gene on chromosome 13 (4), where tryptophan is exchanged to glycine (W524G) at the C-terminal of the protein (5, 6). The corresponding phenotype in the PMN mouse becomes apparent at 2 weeks after birth as progressive paralysis starting in hind limbs. Histological sections showed a subsequent retrograde axonal degeneration of peripheral nerves and loss of motoneuron cell bodies in the spinal cord and brainstem. Respiratory failure at 5 weeks of age corresponds to the relatively early death of the mice (3, 7). On cellular and molecular level, the mutation in the TBCE gene leads to incorrect dimerization of microtubules, which results in an impairment of axonal elongation (5, 8) and coincides with microtubule loss (6). Functional analyses of the auditory system revealed a progressive hearing loss by disturbed or absent auditory brainstem response (ABR) and otoacoustic emissions (OAEs) with a progressive loss of OHCs (9). Primary cultured spiral ganglion neurons showed reduced neurite growth (10). Analysis of the auditory nerve revealed an unaltered quantity of spiral ganglia in the modiolus, but a reduction of microtubules in the axons (9), consistent with expression of modiolus in other mutants (11). Therefore, the PMN mouse is an interesting model for investigating auditory neuropathy.

Several neurotrophic factors (12) and other therapeutic molecules and approaches have been studied to counteract neuronal degeneration in the PMN animal model (8, 13–20). Among them, polyethylene glycol-modified IGF-1 variant (peg-IGF-1) is a stabilized form of IGF-1 (8, 21). The neurotrophic effects of IGF-1 have been shown in various studies and were explained by the prolongation of neuronal survival (22–25) and the enhancement of axonal elongation (26, 27). This prevented muscle atrophy in motor neuropathies (28). In addition, studies have been conducted to test the therapeutic effects of IGF-1 in the sensory epithelium of the cochlea. IGF-1 and its receptor were highly expressed in the organ of Corti during embryonic development, but the levels of expression dropped sharply after birth (29). Knockout of IGF-1 and its receptor resulted in high-level sensorineural hearing loss that is caused by deformation of the otic capsule during embryonic development and disturbed differentiation of the cochlea postnatally (30–32). In addition, the auditory neurons exhibited aberrant innervation, as well as defective myelination (33). Therapeutic local application of recombinant IGF-1 into the middle ear resulted in a reduction of noise-induced hearing loss in gerbils (34). Intratympanic application of recombinant IGF-1 in humans had also been shown to improve threshold after hearing loss refractory to corticosteroid therapy (35, 36). Peg-IGF-1 has been developed by the addition of a polyethylene glycol chain to improve the pharmacokinetics and applicability by reducing rapid clearing through the bloodstream and kidneys and reducing acute side effects by IGF-1. This modification resulted in prolongation of the half-time with full maintenance of the activity (21).

The aim of this study was to investigate the effects of peg-IGF-1 administration for potential otoprotective effects in the PMN mice. Detailed histological examinations of the organ of Corti and electrophysiological investigations of the hearing function were performed. Analysis evidenced structural improvement in efferent synapses and transient OHC rescue, even though without functional benefit. However, further investigations of the organ of Corti during development must be carried out to understand the pathology of the efferent fibers leading to OHC loss in the PMN mouse.

## Materials and Methods

### Animals, Treatment, and Anesthesia

Heterozygous PMN mice (NMRI background) were genotyped as described (8). Animals were fostered and housed under controlled conditions (20–22°C, 55–65% humidity, 12:12-h light/dark cycle, food and water *ad libitum*) and received injections of 0.15 mg/kg body weight peg-IGF-1 [Roche, Basel (21)] subcutaneously from postnatal day (p) 15 on every second day. Mice were controlled for vitality and health daily. All mice used for audiometry were carefully matched for age and sex to minimize the confounding effects on the measurement outcomes. The experimental procedures were performed according to German regulations on animal welfare in agreement with and under control of the local veterinary authority and Committee on the Ethics of Animal Experiments (license number 55.2-DMS-2532-2-80).

Audiological examinations were performed in homozygous PMN and homozygous wildtype mice (NMRI) at the age of postnatal days 21 and 28. For anesthesia, a mixture of ketamine hydrochloride (75 mg/kg body weight, Ketavet 100, Pharmacia) and xylazine hydrochloride (5 mg/kg body weight, Rompun 290, Bayer) was injected intraperitoneal with an injection volume of 1 ml/kg body weight. Anesthesia was maintained by application of 10% of the initial dose, typically in 30-min intervals. Body temperature was maintained by a temperature-controlled heating pad to 37°C.

### Audiometric Assessment

Hearing function assessment was performed *via* Tucker-Davis Technologies Inc. (TDT, USA) setup using the programming SigGenRZ software and the TDT BioSigRZ performing platform. The equipment loudspeaker control, microphone (378C01, PCB Pieztronics Inc., N.Y., USA), acquisition, processing, averaging, and data management were further coordinated using the RZ6 Multi I/O Processor System.

### Distortion Product Otoacoustic Emissions

A total of two pure tones (f1 and f2, f2/f1 = 1.2) were used to measure distortion product otoacoustic emissions (DPOAEs). The primary tone f1 is 0.894 times and the primary tone f2 is 1.118 times the fundamental frequency. Through a low noise recording microphone, primary tones produced by two separate loudspeakers were introduced into the closed ear canal of the animal and emissions were recorded. If a peak in the spectrum at 2(f1–f2) exceeded the background noise by 3 dB μV, then it was defined as a positive DPOAE. Fixed test frequencies ranged from 4 to 24 kHz in 4kHz steps and a stimulus level of 90 to 30 dB sound pressure level (SPL) in 5 dB steps were performed and averaged from 128 responses. DPOAEs were always performed prior to ABR, since the reverse procedure can result in a temporary reduction of DPOAEs (37).

### Auditory Brainstem Response

For recording of monaural bioelectrical auditory brainstem potentials, subdermal stainless-steel electrodes (27GA 12 mm, Rochester Electro-Medical, USA) were inserted subcutaneously to the ventrolateral side of the ear (active), on the vertex (negative), and on the body (neutral) and connected to a preamplifier (RA4PA, TDT, USA) with 20-fold amplification. To verify proper electrode positioning or conductivity, impedance measurements of all electrodes (<5 kΩ) were taken prior to each ABR recording. The bioelectrical ABR signals recorded from the subdermal electrodes were transferred to a head stage (RA4LI, TDT, USA). The sounds were applied *via* a microphone that was inserted into the external auditory canal (active ear). For ABR threshold recordings and wave latency, click stimuli and tone bursts between 4 and 24 kHz were applied. The stimuli started at a sound pressure level of 90 dB and decreased in 5 dB steps to 30 dB. The responses were amplified 20-folds and filtered with a bandpass filter set at 0.3–3.0 kHz. Responses were sampled over a 10-ms period and averaged from 512 responses to determine the minimum threshold. The responses were sampled over a period of 10 ms and averaged from 512 responses. The minimum threshold was assumed to be the last wave that could still be reproduced. Wave I represents the distal part of the auditory nerve, whereas wave II portions the proximal of the auditory nerve (AN) in the cochlear nucleus (NC) in the brainstem. The superior olivocochlear (SOC) is presented by wave III, respectively. Waves IV and V are produced by evoked neural responses in the inferior colliculus (IC) and the nucleus lemniscus lateralis (NLL) (38, 39).

### Tissue Preparation and Whole-Mount Dissection

Mice were deeply anesthetized with CO2 and intracardially perfused with 4% PFA in 1M PBS pH 7.4 by the force of gravity. After decapitation and removing the brain, the temporal bones were identified and the cochleae were encapsulated, post-fixated for 1 h and decalcified in 125 mM EDTA overnight on a 3D rotator at room temperature. Once decalcification was complete, the cochleae were stored in 1M PBS pH 7.4 until use or immediately dissected for whole-mount staining. Cochleae were simultaneously stained, embedded, and imaged microscopically at the same conditions.

### Immunohistochemistry

After dissection, cochlear turns were separated in a 24-well plate submerged with 1% PFA in 1M PBS pH 7.4. For the following steps, the tissue was always covered in liquid and rotated on a 3D incubator. After blocking or permeabilization with 10% normal horse serum (NHS), 1% bovine serum albumin (BSA), 1% Triton X100, and 0.1% Tween20 in 1M PBS pH 7.4 for 1 h, the cochlear tissue was incubated with antibody solution (3% NHS, 1% BSA, 0.3% TritonX100, 0.1% Tween20) containing primary antibodies against ChAT (anti-rabbit, PA529653, Invitrogen, 1:1000) and ßIII-tubulin (anti-mouse, MAB1192, R&D System, Tuj1, 1:1000) for 3 h at room temperature. Cochlear turns were washed three times with 0.3% TritonX100 and 0.1% Tween20 in 1M PBS pH 7.4. Primary antibodies were detected with secondary Alexa-555 (anti-rabbit, Invitrogen, A32794, 1:1000) or Alexa-647 (anti-mouse, Invitrogen, A32773, 1:1000) conjugated antibodies for 1 h at room temperature. In addition, labeling with phalloidin-conjugated Alexa-488 (A-12379, Invitrogen, 1:800) was performed.

### Confocal Microscopy

Immunolabeling was analyzed using an Olympus IX81 microscope equipped with an Olympus FV1000 confocal laser scanning system, an FVD10 SPD spectral detector, and diode lasers of 473, 559, and 651 nm. The images were acquired with an Olympus UPLSAPO 40X objective (oil, numerical aperture: 1.3). For high-resolution confocal scanning, a pinhole aperture setting was used, which represented a diffraction disk. Whole-mount images of the organ of Corti were taken in about 300-nm steps in the *z*-axis. Z-stacks, brightness, and contrast of the images were adjusted using ImageJ for better visualization.

### Data Analysis and Statistics

The values of the DPOAE measurements were converted before analysis. The RZ6 processor is a +/– 10V device, and therefore, the described levels of the TDT system are inherently interpreted as dB Volt (dBV). The following equation after TDT is used to convert dBV to dB SPL:


$$\begin{array}{l} 20log\;\left(\frac{10x^{\left(\frac{dBV}{20}\right)}\;}{0.05}\right)\;+\;93.9 \end{array}$$

Hearing thresholds of DPOAEs are defined as the last positive SPL value. Outer hair cell function at 8 kHz was assessed by linear regression of the data points and DP-gram of 4, 8, 12, 16, 20, and 24 kHz.

Auditory brainstem response thresholds were set at the last reproducible waveform. Values of all animals of each group were averaged and are presented as hearing thresholds ± standard errors of mean, respectively. ABR analysis included latency and the maximum height of wave I and II of click ABR.

Examination of outer hair cells and ßIII-positive neurons was performed with ImageJ in portions of 100 μm. Analysis of the cholinergic synapses of the MOC fibers was evaluated *via* choline acetyltransferase (ChAT) immunostaining with ImageJ ROI manager. Confocal images were 16-bit images with 1,024 pixel × 1,024 pixel in 317.44 μm × 317.44 μm. The synapse area is defined and calculated by the number of contained pixels divided by 3.23 pixel/μm. The mean gray value is the average of the values of all pixels divided by the number of pixels of the selected area. The gray scale range is from 0 (black) to 4,095 (white), where the latter was expected as saturated. Colors were set virtually for visualization.

For quantification of synapse entropy, the center of the synapses was manually annotated with ImageJ. Based on graph theory, neighboring synapses were connected to create a weighted undirected cyclic graph. For each annotated synapse, the angles between the connection were determined. Synapses with good organization should have angles close to (or multiple of) 90 degrees. Therefore, the mean absolute deviation (MAD) from 90 degree was determined for each image as a measure of synapse entropy. The analysis was performed with python. Data were normal distributed, as tested with the Shapiro test, and analyzed for significant changes with the *t*-test.

The remaining statistical analysis and graphic display were performed in GraphPad Prism software (GraphPad, San Diego, CA, USA). A column analysis was performed to determine normal distribution. In the case of a Gaussian distribution, an ANOVA and Tukey's multiple comparison *post hoc* test or Holm-Sidak's multiple comparisons test was performed, where mean and standard error of the mean (SEM) were determined. A *p*-value of <0.05 was considered as significant. Significances were always determined by comparison with wildtype animals, if not otherwise noted. All data are presented in mean ± SEM unless otherwise noted. Wildtype mice are illustrated in black (*n* = 7), untreated PMN mice in dark red (*n* = 4), and treated PMN mice (*n* = 4) with 0.15 mg/kg peg-IGF-1 in light red for p21 and p28. Final processing of images was performed with ImageJ and Photoshop CS9 (Adobe).

## Results

### Synapses Formation and Intensity of Cholinergic Acetyltransferase

**Figure 1:**
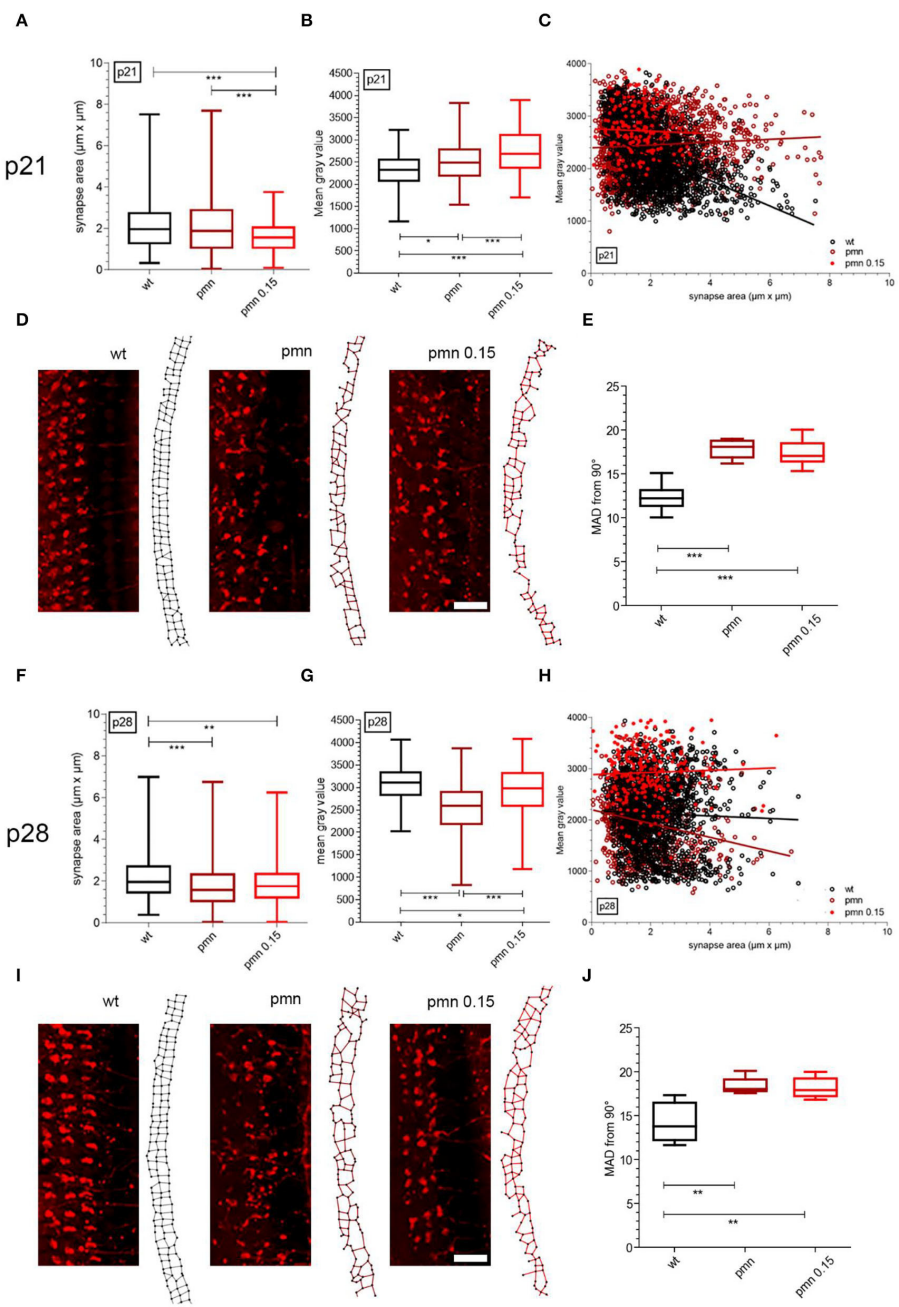
Evaluation of cholinergic synapses of MOC fibers at p21 and p28 with peg-IGF-1 treatment. **(A)** Analysis of the synapse area and **(B)** the mean gray value demonstrated a significantly decreased area and an increased mean gray value of treated PMN animals compared to untreated PMN littermates and wildtype mice (*p* < 0.001). In addition, PMN animals showed an increasing mean gray value compared to the wildtype (*p* < 0.01). **(C)** The ratio of synapse area and mean gray value of treated PMN animals were different compared to untreated and wildtype mice. **(D)** Examples of p21 cholinergic synapses and a representative synapse graph at the OHCs of wildtype, PMN and treated PMN mice with 0.15 mg/kg peg-IGF-1. **(E)** Quantification of synapse entropy indicates significantly disturbed synapse alignment of untreated and treated PMN mice (*p* < 0.001). **(F)** PMN animals had a significantly decreased synapse area compared to wildtype littermates (*p* < 0.001), but at this age **(G)**, untreated PMN mice had a decreased mean gray value of ChAT intensity to treated PMN animals and the wildtype (*p* < 0.001). **(H)** Each group pointed out a different ratio of synapse area to mean gray value of ChAT (*p* < 0.001). **(I)** Examples of p28 cholinergic synapses scanned by confocal microscopy and a representative synapse graph of wildtype, PMN and treated PMN mice with 0.15 mg/kg peg-IGF-1. Immunohistochemical analysis suggests regression of synaptic organization in treated PMN animals. **(J)** However, values of synapse entropy evaluation depict still significantly disturbed alignment (*p* < 0.01), Synapse area, mean gray values: Ordinary one-way ANOVA with multiple comparison test and the *post hoc* Holm-Sidak analyses. Scale bar: 10 μm. Significances: *p < 0.05, **p < 0.01, ***p < 0.001.

Outer hair cells are primarily innervated by efferent cholinergic and afferent non-cholinergic nerve fibers. Since PMN mice undergo a severe OHC loss, the presynapses of these neurons were examined *via* ChAT staining. At p21, the area of the OHC synapse of treated PMN mice (1.58 ± 0.06 μm^2^) was significantly (*p* < 0.001) smaller compared to the wildtype (2.13 ± 0.02 μm^2^) and untreated PMN littermates (2.12 ± 0.03 μm^2^) (Figure 1A). The mean gray value of the synapse (2,728 ± 36) of peg-IGF-1-treated PMN mice was significantly (*p* < 0.001) higher compared to wildtype (2,455 ± 14) and untreated PMN mice (2,231 ± 13). Additionally, the mean gray value of PMN mice was significantly (*p* < 0.05) higher compared to the wildtype (Figure 1B). In the linear plot of area to mean gray value of MOC fiber synapses, the larger average synapses of PMN mice (slope: 8.71 ± 3.37) expressed a higher value of ChAT than synapses of the same size in wildtype mice (slope: −89.06 ± 4.79), which expressed a decreasing value of ChAT (Figure 1C). Treated PMN mice had smaller synapses in comparison, but the trend shows that a reduced ChAT intensity is to be expected with larger synapses (slope: −14.14 ± 6.97). The different arrangement of efferent synapses of PMN animals and control animals was striking (Figure 1D). To quantify synaptic entropy, we generated plots in which adjacent synapses were connected (Figure 1D). The mean absolute deviation (MAD) of 90-degree angles between synapses of treated and untreated PMN mice is significantly increased compared with wildtype mice (*p* < 0.001) (Figure 1E).

At p28, ChAT-positive synapses of peg-IGF-1-treated (1.9 ± 0.07 μm^2^) and untreated PMN mice (1.79 ± 0.02 μm^2^) were significantly smaller (*p* < 0.01) compared to the wildtype animals (2.15 ± 0.02 μm^2^). There was no significant difference between treated and untreated PMN mice (Figure 1F). The mean gray value of the OHC synapse of PMN mice (1,960 ± 15) was significantly (*p* < 0.001) lower compared to the wildtype (2,127 ± 16) and treated PMN mouse littermates (2,927 ± 36) (Figure 1G). The synapses of the treated PMN mouse had an overall high proportion of ChAT (slope: 19.84 ± 11.33), which was also seen in the large synapses. Untreated PMN mice showed a strong tendency of low ChAT values as the size of the synapse increases (slope: −64.14 ± 7.83). In contrast, linear regression showed an almost equal amount of ChAT in the synapses of the wildtype mice regardless of size (slope: −9.8 ± 5.11) (Figure 1H). Immunohistochemical analysis pointed out a partially restored alignment of synapses (Figure 1I), but the MAD of PMN mice was still highly disorganized (*p* < 0.01) (Figure 1J).

### Immunohistological Evaluation of the Cochlea—Outer Hair Cells and Neuronal Fibers

**Figure 2:**
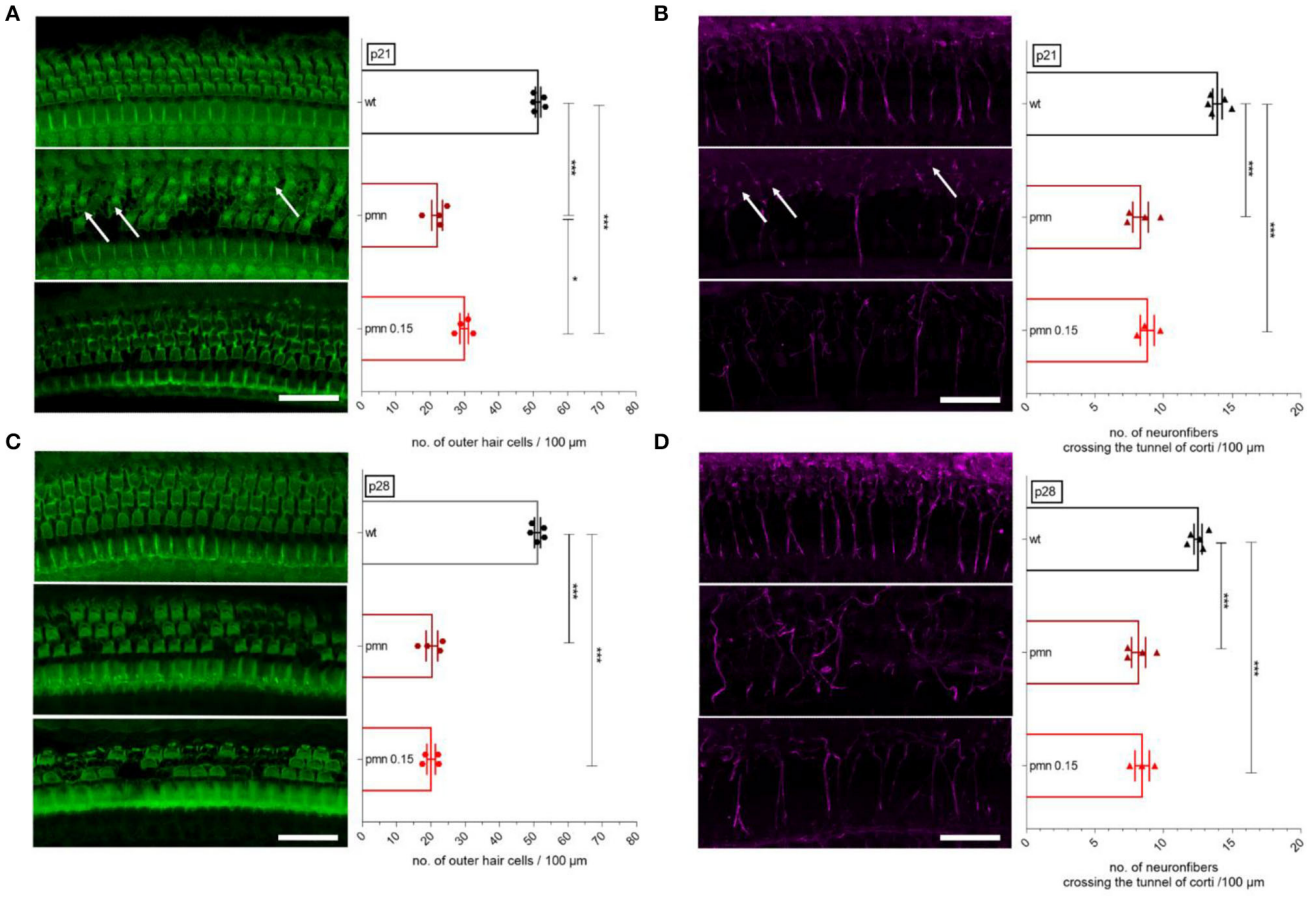
Whole-mount staining and evaluation of the organ of Corti. Examples of immunostaining show the OHCs (green, F-actin) and neuronal fibers crossing the tunnel of Corti (magenta, ßIII-tubulin). **(A)** At p21, the number of OHCs was significantly reduced in PMN animals (*p* < 0.001). Interestingly, peg-IGF-1 has a positive effect on the OHCs compared to the untreated PMN littermates (*p* < 0.05). **(B)** At the same age, neuronal fibers in the tunnel of Corti were significantly reduced in all PMN groups (*p* < 0.001). Here, peg-IGF-1 showed no effect. Although the neural fibers were reduced, synapses and OHCs were still present (white arrow). **(C)** At P28, treated and untreated PMN animals had the same decreased amount of OHCs (*p* < 0.001). **(D)** Regarding neuronal fibers, all PMN mice had fewer neuronal fibers (*p* < 0.001). Ordinary one-way ANOVA with multiple comparison tests and the *post hoc* Tukey analysis. Scale bar: 20 μm. Significances: *p < 0.05, ***p < 0.001.

Based on the beneficial impression of peg-IGF-1 on MOC synapses, the morphology of the organ of Corti was processed by immunohistochemistry. At the age of p21, wildtype animals had 52 ± 1 OHCs per 100 μm, which were significantly (*p* < 0.001) reduced in untreated (22 ± 2) and treated (30 ± 1) PMN littermates. A significantly (*p* < 0.05) higher number of OHC was seen in treated PMN mice compared to untreated littermates (Figure 2A). In the wildtype animals, 13 ± 1 neuronal fibers crossed the tunnel of Corti, which were significantly (*p* < 0.001) reduced in untreated (8 ± 1) and in treated (9 ± 1) PMN animals. However, synapses and OHCs of PMN animals were still present with absent axonal connection (white arrow) (Figure 2B).

At p28, 51 ± 2 OHCs per 100 μm were counted in wildtype mice, whereas in PMN mice without peg-IGF-1 application (20 ± 2) and in treated PMN mice (20 ± 1), a significantly (*p* < 0.001) reduced number of OHCs per 100 μm was analyzed (Figure 2C). A number of 13 ± 1 per 100 μm neuronal fibers crossing the tunnel of Corti were counted in wildtype mice. Treated and untreated PMN littermates had a significantly (*p* < 0.001) reduced number of fibers (both 8 ± 1) crossing the tunnel of Corti (Figure 2D).

### DPOAEs– Thresholds and Linear Regression

**Figure 3:**
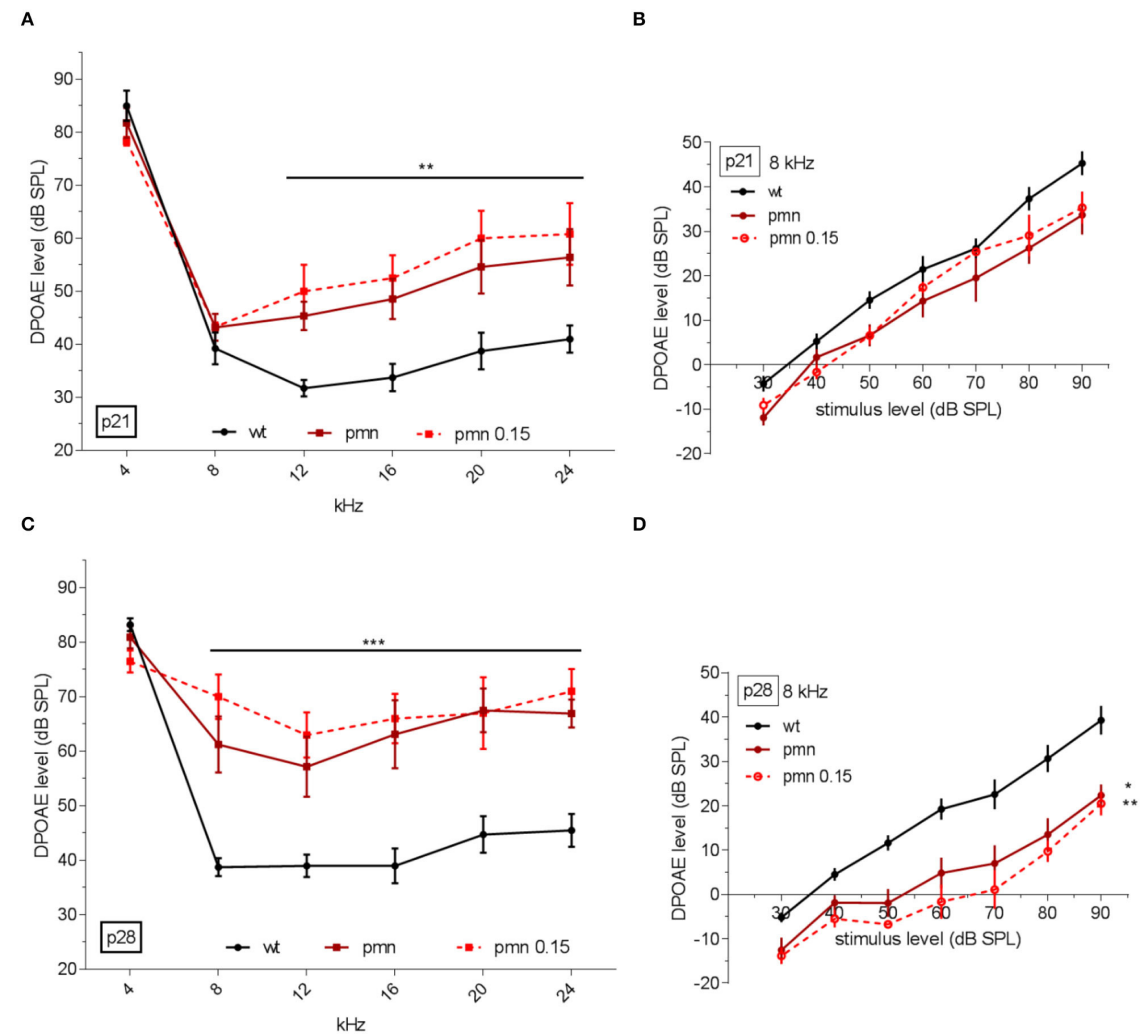
DPOAE analysis in dB SPL at recorded frequencies. **(A)** DPOAE recorded hearing threshold at p21. Treated and untreated PMN mice had a significant increase at 12, 16, 20, and 24 kHz (*p* < 0.01) compared to the control littermates. **(B)** A slope function at 8 kHz showed no significantly different slope function between the three groups. **(C)** At p28, PMN mice had an increased threshold at 8, 12, 16, 20, and 24 kHz (*p* < 0.001). **(D)** At this age, the slope function showed a significant reduction in untreated PMN (*p* < 0.05) and treated PMN mice (*p* < 0.01) compared to the wildtype animals. DPOAE thresholds: Regular two-way ANOVA with multiple comparison tests and *post hoc* Tukey analysis. Slope function: Ordinary one-way ANOVA with multiple comparison tests and Holm-Sidak post hoc analysis. Significances: *p < 0.05, **p < 0.01, ***p < 0.001.

Since peg-IGF-1 had a positive effect on cellular levels in synapses of MOC fibers by ChAT immunolabeling and delayed temporary OHC loss, the question arose whether the hearing performance would improve under peg-IGF-1 treatment. For this purpose, otoacoustic emissions were evaluated to investigate the electromotility of the OHCs. At p21, PMN mice with or without peg-IGF-1 treatment did not exhibit significant differences in DPOAE thresholds compared to controls at 4 and 8 kHz. Independent of peg-IGF-1 treatment, PMN mice showed a significantly increased DPOAE threshold at 12, 16, 20, and 24 kHz (*p* < 0.01) (Figure 3A). No differences between treated and untreated PMN mice were detected by DPOAE measurements. At 8 kHz, no significant different slope function was observed between the three groups (Figure 3B). At this age, peg-IGF-1 had no effect on the DPOAE level of PMN mice.

By the age of p28, all PMN mice showed significantly increased DPOAE threshold compared to wildtype mice at 12, 16, 20, and 24 kHz, and now also at 8 kHz (*p* < 0.001) (Figure 3C). The data showed no difference between treated and untreated PMN mice. However, PMN treated (*p* < 0.01) and untreated mice (*p* < 0.05) had a significantly lowered slope of DPOAEs at 8 kHz compared to the wildtype animals (Figure 3D). Taken together, the data suggested no effect of peg-IGF-1 on the DPOAE level of PMN mice.

### Click and Frequency-Specific ABR Thresholds

**Figure 4:**
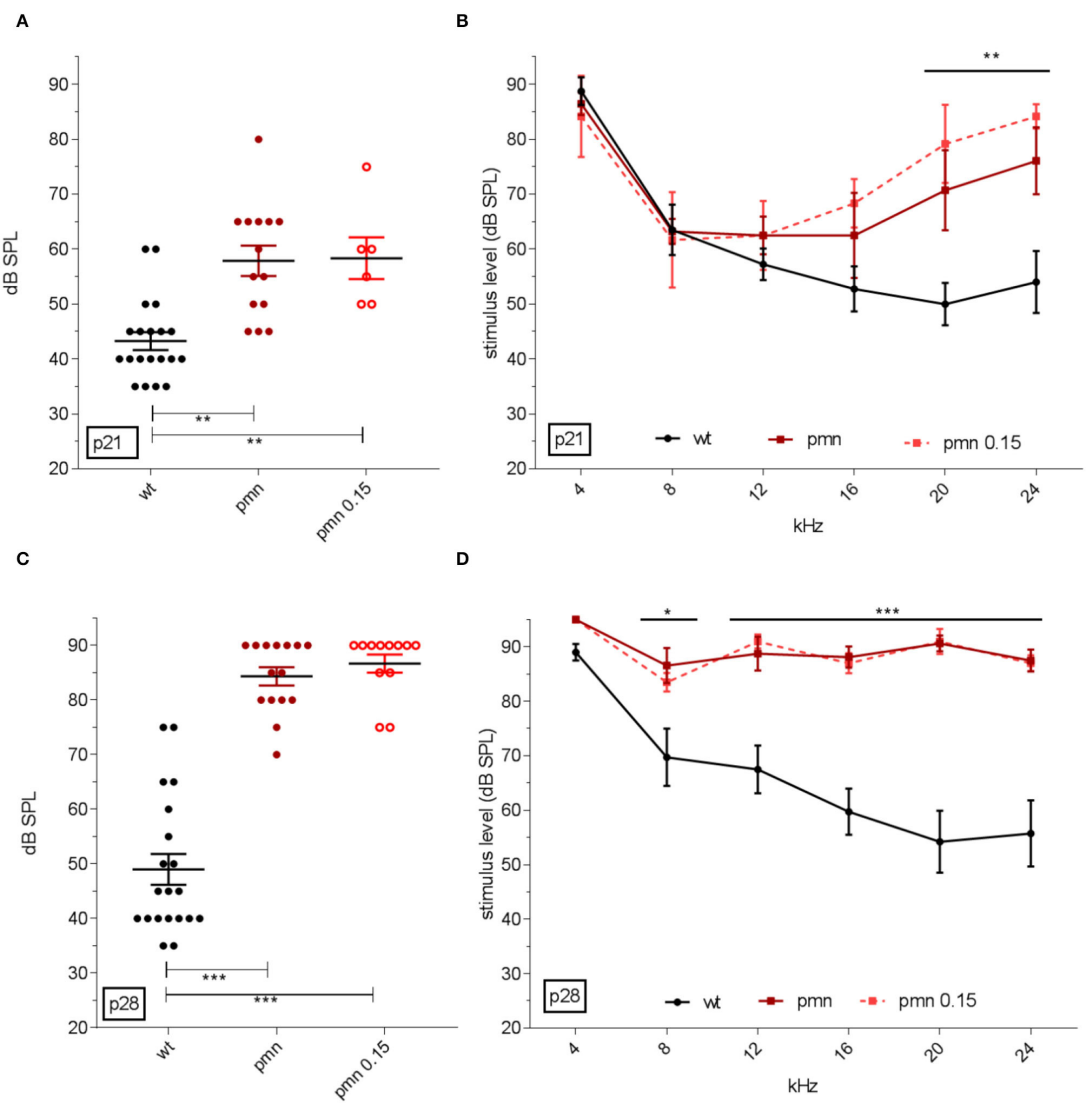
ABR click and tone hearing threshold. **(A)** Hearing threshold of click ABR and **(B)** frequency-specific thresholds at p21 in dB SPL. Click ABR showed an increased threshold in PMN mice (*p* < 0.01) and tone-specific ABR indicated an increased threshold at the higher frequencies of 20 and 24 kHz (*p* < 0.01). Peg-IGF-1 had no benefit on the click and tone-specific ABR. Similar results could be seen in **(C)** the threshold of click ABR and **(D)** frequency-specific thresholds at p28. Click ABR analysis showed an increased threshold of both PMN groups (*p* < 0.001) and an increasing threshold at the frequencies 8 (*p* < 0.05), as well as 12, 16, 20, and 24 kHz (*p* < 0.001). Click ABR threshold: Ordinary one-way ANOVA with multiple comparison test and Hold-Sidak post hoc analysis. Tone ABR: Regular two-way ANOVA with multiple comparison tests and Tukey post hoc analysis. Significances: *p < 0.05, **p < 0.01, ***p < 0.001.

In addition, ABR analysis was performed to investigate hearing threshold. At p21, click-ABR threshold in wildtype animals was elicited at 43 ± 2 dB SPL. PMN mice showed an ABR threshold at 58 ± 2 dB SPL. PMN mice treated from p14 to p21 with peg-IGF-1 showed an ABR threshold at 59 ± 3 dB SPL (Figure 4A), indicating that this treatment did not change this threshold. All PMN mice had a significantly (*p* < 0.01) higher ABR threshold compared to control littermates. Frequency-specific evaluation showed a significant (*p* < 0.01) increase in ABR thresholds at 20 and 24 kHz in treated and untreated PMN mice compared to wildtype (Figure 4B). The treatment had no effect on ABR-thresholds in PMN mice at this age.

At p28, the click-ABR threshold of wildtype mice was 46 ± 3 dB SPL, whereas ABR thresholds of PMN mice further increased to 84 ± 2 dB SPL, and in peg-IGF-1-treated PMN mice at 85 ± 2 dB SPL (Figure 4C). Again, all PMN mice had a significantly (*p* < 0.001) higher ABR threshold compared to the wildtype littermates. Both untreated and treated PMN mice had a significantly (*p* < 0.05) higher ABR threshold at 8 kHz, and at 12, 16, 20, and 24 kHz (*p* < 0.001) compared to wildtype animals (Figure 4D). These data indicate that peg-IGF-1 treatment had no effect on the hearing level of PMN mice.

### Neuronal Latency and Amplitudes of the Auditory Nerve

**Figure 5:**
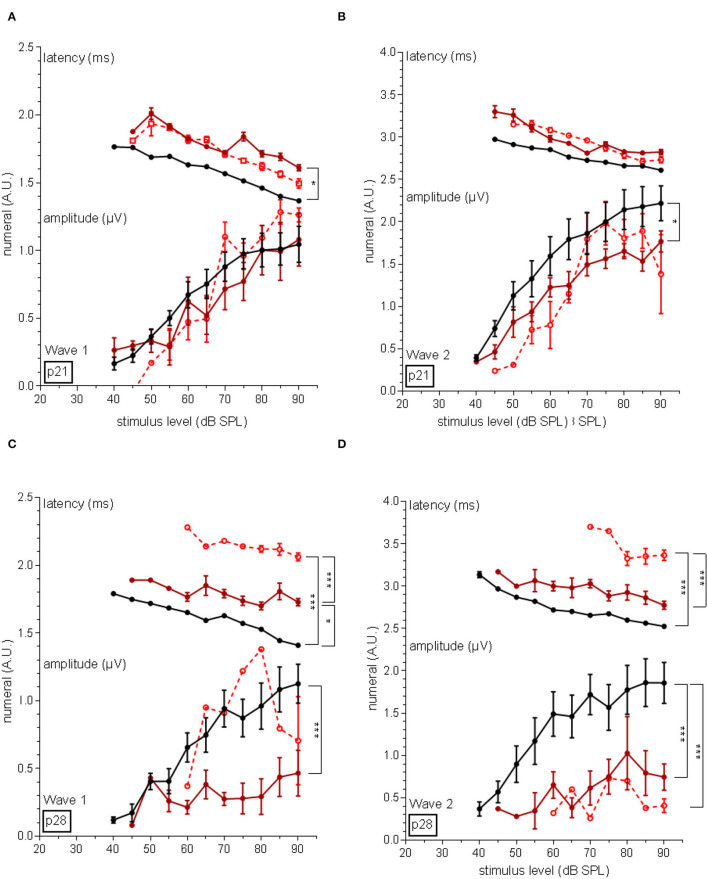
Amplitudes and latency of wave I and II of the auditory nerve. **(A)** At the age of p21, wave I of untreated PMN mice showed a latency shift compared to the wildtype animals (*p* < 0.05). Amplitudes of PMN mice did not differ. **(B)** 1 week later, untreated PMN mice still implicated a latency shift (*p* < 0.05) but treated PMN mice displayed an increased latency compared to wildtype and untreated PMN littermates (*p* < 0.001). Only untreated PMN animals showed a decreased amplitude of wave I at p28 (*p* < 0.001). **(C)** Regarding wave II, the latency did not differ between the mice, but in untreated PMN animals, a decreased amplitude at the age of p21 (*p* < 0.05) was recorded. **(D)** At p28, treated PMN mice displayed a significant latency shift compared to the untreated PMN and wildtype animals (*p* < 0.001). The amplitude of wave II of both PMN groups was decreased (*p* < 0.001). Ordinary one-way ANOVA with multiple comparison tests and Tukey post hoc analysis. Significance relations are indicated with clips. Significances: *p < 0.05, ***p < 0.001.

Furthermore, amplitudes and latencies were assessed of distal and proximal regions of the auditory nerve. The latency of wave I in p21 PMN mice was significantly longer (*p* < 0.05) compared to the wildtype mice, but no different amplitude was recorded (Figure 5A). After 1 week, the latency of wave I of PMN mice was also significantly (*p* < 0.05) longer, but also treated PMN mice had a significantly (*p* < 0.001) increased latency compared to the wildtype littermates and untreated PMN mice (Figure 5B). In addition, in untreated PMN mice, a significantly (*p* < 0.001) lower amplitude of wave I compared to wildtype littermates was recorded.

Regarding wave II at p21, PMN mice had no latency shift, but a significantly (*p* < 0.05) lower amplitude, compared to the wildtype and untreated PMN littermates (Figure 5C). At p28 in both PMN groups, a significantly (*p* < 0.001) increased latencies and decreased amplitudes were evaluated compared to the wildtype animals (Figure 5D).

## Discussion

Progressive motor neuropathy mice are a model of hearing loss characterized by an auditory neuropathy accompanied by loss of outer hair cells (9). These mice have been primarily described as a model of motor neuropathy with progressive dysfunction and loss of spinal motoneurons (9). A previous study has shown that peg-IGF-1 treatment can improve the motoric symptoms in this animal model (8). IGF-1 belongs to the insulin family of proteins, which controls cell proliferation, differentiation, and cell survival. The effects are mediated through PI3K/Akt and MEK/ERK pathway activation, which also were exhibited in cells of the sensory epithelium in the inner ear (40, 41). In the past, IGF-1 application has demonstrated protective effects on hair cells (34, 35, 41–43). Moreover, clinical trials have shown that IGF-1 is also effective in treatment for sensorineural hearing loss in humans (36, 44, 45). A stabilized form of IGF-1 was generated by the addition of a 40 kDa polyethylene glycol chain to lysine 68. This modification caused a prolonged half-life of the agent in the blood, resulting in detectable increased levels in the periphery and brain, and additionally reduced hypoglycemia in humans (21, 46). Previous study of peg-IGF-1 in PMN mice showed enhanced survival, higher body weight, greater axon length, higher neuron number, improved motor coordination, and raised muscle force (8). The positive results of the above studies raised the hypotheses that peg-IGF-1 could also have an otoprotective effect on the auditory pathology of the PMN mice.

### Positive Otoprotective Effects of peg-IGF-1 on the Innervation of the Outer Hair Cells

Cholinergic efferent neuron fibers represent 95% of the axons crossing the tunnel of Corti (47, 48). The rest consists of type 2 spiral ganglion neurons (49, 50). Therefore, OHCs are mainly innervated by myelinated MOC fibers, which belong to the descending neural auditory pathway (47). Since PMN mice showed a progressive outer hair cell loss, the innervation of OHCs was further examined. This pathway begins at the superior nucleus olivaris of the medulla oblongata and divides into the lateral pathway (LOC: lateral olivocochlear) by innervating inner hair cells, and the medial (MOC: medial olivocochlear) pathway, which innervates exclusively outer hair cells by efferent cholinergic neurons (51–53). The MOC synapses were analyzed *via* ChAT immunostaining.

At p21, the synaptic area of PMN animals was not significantly different to control animals, but the range of the area's size was enhanced, probably reflecting a disorganization of the synapse. In addition, there was a higher ChAT intensity compared to the control animals. The treatment of peg-IGF-1 resulted in comparison with the other two groups in a reduction of the synapse area, but also to an enhancement of the ChAT intensity. At p28, both PMN groups showed a reduction of the synaptic area, with a reduced ChAT activity in the untreated PMN mice, but a rescue to normal level by peg-IGF-1 treatment. However, the alignment of synapses of treated and untreated PMN mice was significantly disturbed at both time points.

The positive effect of peg-IGF-1 might be explained by the fact that IGF-1 stimulates exocytosis in presynapses by activation proteins of the SNARE complex (54–57), especially synaptotagmin 10 (56). Therefore, IGF-1 was already used therapeutically in several models of sensory defects, for example, in the visual or olfactory field. In these studies, IGF-1 also induced a regrowth and formation of axons and their terminals, but in contrast to our study, it was not able to recover the sensory function (58, 59).

Reduced ChAT intensity was depicted before in facial neurons of the PMN mice (18), which are induced by axonal swelling (5) and slowed retrograde axonal transport (18, 60). Axonal swellings, called spheroids, are defined as local and timely varicosities in axons containing disorganized filaments and deformed mitochondria due to the TBCE mutation (60). In this work, we could show disorganized synapse alignment, too. However, peg-IGF1 was not able to restore the rganization of efferent synapses. Spheroids were found in long sensory axons repeatedly starting distal. Thus, sensory nerve fibers demonstrated multifocal degeneration processes such as the motor neurons of the PMN mouse (2, 60).

A loss of neurons crossing the tunnel of Corti was detected at p21 and p28. The numbers of neuronal fibers in the auditory nerve depicted no beneficial improvement by peg-IGF-1 treatment. The loss of axons in the tunnel of Corti can be explained by the degeneration of MOC fibers. These neurons, which are the longest in the auditory pathway, are affected by the TBCE mutation in PMN animals, especially at their peripheral synapses (5, 6). Similar findings have been reported in Wobbler mice, another model of progressive neuronal degeneration (61). These mice also have a defective axonal transport (62) and a progressive axonal degeneration (63) due to the downregulation of the tubulin chaperone TBCA and α3-tubulin (64).

The defect in the MOC fibers can be described as a special phenotype of auditory neuropathy, thus termed “efferent auditory neuropathy.” This phenotype has been rarely shown before. According to our knowledge, there is only one described mouse model, the ISLET1 transgenic mice, which is also characterized by an altered cholinergic efferent innervation of the OHC with decreased volume of ChAT in its terminals (65). Further studies are needed to fully prove an efferent neuropathy. In this regard, afferent neuropathy should be refuted by immunohistochemical analysis with appropriate synaptic markers (66). Furthermore, *in vivo* and/or *in vitro* electrophysiological experiments on the auditory nerve are required (67).

### Attenuation of Outer Hair Cell Loss Without Functional Effect by peg-IGF-1 Treatment

Since peg-IGF-1 treatment leads to a positive effect on the synapsis of the OHCs, their function was additionally investigated by distortion product otoacoustic emissions (DPOAEs), which are direct electrophysiological responses of the outer hair cells. The DPOAE thresholds of p21 PMN mice were higher than the control group. A further increase of DPOAE thresholds was detected at p28 in both PMN groups compared to the wildtype. In addition, the number of OHCs was similarly reduced at both ages in PMN animals, but the increasing DPOAE thresholds at p28 indicated that there is also a loss of function in the remaining OHCs. Interestingly, peg-IGF-1 had a significant positive influence on the number of the outer hair cells at p21, but no effect on the DPOAE threshold. At p28, the positive effect of peg-IGF-1 on the numbers of outer hair cells was dismissed.

In a previous study (9), similar pathology regarding the number of OHC and the DPOAEs was shown in the PMN mouse. This contrasts to most of the auditory neuropathy models, which are normally characterized by detectable OAEs, due to the persistent function of the OHCs (67, 68). However, some mouse models for auditory neuropathy have also shown DPOAE abnormalities due to sporadic OHC loss or dysfunction of the cochlear amplifier (69–71), especially in the case of synaptic disorders (65, 72). The PMN mouse, which is primarily described as a solely neuropathy model based on microtubule disorders in the peripheral nervous system (2, 3), represents a new type of mouse models for auditory neuropathy, characterized by a more pronounced progressive OHC loss compared to the classical auditory neuropathy models.

A transient otoprotective effect of peg-IGF-1 was seen at p21. Similar effects of IGF-1 have been demonstrated before in *in vitro* experiments of cochlear explants (73–75). The effect was explained by the activation of the supporting cells in the organ of Corti, since the IGF-1 receptor is highly expressed in these cells (42). Protein phosphorylation of downstream targets of IGF-1 might also play an important role, as it has been shown in other tissues (76). Phosphorylated (p-)AKT was found on the inner sulcus cells, whereas p-ERK was activated in Claudius and Hensen's cells, which are located close to the OHCs (42). However, how the phosphorylation of PI3K/Akt and MEK/ERK in the supporting cells contribute to the otoprotective effect of IGF-1 on the hair cells is still unknown (77). Furthermore, other neurotrophic factors were tested in animal models of hearing loss. For example, NGF or NT3 application prevents the OHC loss in mouse models of early-onset progressive hearing loss (78–80).

### Latency Shift and Decreased Amplitude With peg-IGF-1 Treatment

Different waves in the ABR spectrum reflect sound stimulation-evoked neural responses in different parts of the auditory pathway. A latency shift of untreated PMN mice were measured at p21 in wave I, but amplitudes of the animals showed no altered values. At p28, peg-IGF-1 treated PMN mice had a latency shift of wave I compared to untreated and wildtype animals. The latency of untreated PMN mice was also shifted compared to wildtype animals. Amplitudes of wave I were reduced in treated animals. Wave II measurements showed amplitude discrepancies of untreated PMN compared to the wildtype and treated PMN mice at p21 with unaffected latency values. At p28, treated and untreated PMN mice had decreased amplitude values and a latency shift in ABR wave II.

Alterations in electrophysiological properties have been described in a previous study in the facial and the ischiatic nerve of the PMN mice (7). These were explained by the subsequent retrograde axonal degeneration of the peripheral neurons characterized by prominent axonal caliber irregularities and axonal swelling with spheroids and formation of myelin ovoids, small cellular chambers containing myelin debris and disrupted axons (60). Disturbed TBCE function impaired microtubule-dependent axonal transport. Similar morphological findings have also been displayed in other studies of neurodegenerative diseases (81–83) accompanied by electrophysiological alterations such as latency shifts and altered amplitudes like in *Map1b* heterozygous mutation mice (84).

The pronounced latency shift of peg-IGF-1-treated mice was also detected in a previous study in the phrenic nerve of PMN mice (8). This effect might be explained by the distinct (side) effect of IGF-1 to slow down neuronal communication (85, 86). This can be explained by the stimulation of the number of myelinated axons and the thickness of myelin sheaths (87–89), which is induced by stimulating myelin-specific protein gene expression like protein zero (89, 90) promoting oligodendrocyte proliferation, neuronal outgrowth, and survival (91, 92). Consequently, the thickened myelin might be the cause of the electrophysiological changes.

### No Effect on the ABR Thresholds by peg-IGF-1 Treatment

Auditory brainstem responses (ABR) are EEG signals in response to an auditory stimulus. In this study, the thresholds of ABR recordings of treated PMN mice showed no significant differences compared to untreated PMN mice. Both PMN groups showed a hearing loss in the high frequencies (20 and 24 kHz) at p21 which proceeded to a complete hearing loss over all recorded frequency spectrum at p28. Peg-IGF-1 was not able to prevent the progressive hearing loss or improve the auditory function.

A comparable progressive course of hearing loss was detected in PMN animal in a previous study (9). In addition, there are other mice models of auditory neuropathy, which also exhibited a similar progression of ABR thresholds (67, 93). These mouse models can be divided into subcategories. One group includes abnormal myelination (94–98), whereas others show complete loss of SGN (99, 100) or contain dysfunctional neurons (70, 84). This should be investigated in additional examinations. Another important category of neuropathy includes regulation of synaptic transmission (101–104).

Neurotrophic factors such as BDNF, CNTF, NT3, and NGF have been tested in animal models of auditory neuropathy in different studies (105). A single application of these factors leading to an overexpression of factors had no effect on ABR threshold (106, 107). Interestingly, simultaneous application of neurotrophic factors together with electrical stimulation has been shown to be otoprotective by improving ABR thresholds (108, 109). This combination might be beneficial in the treatment of hearing loss.

## Conclusion

This study described for the first time that peg-IGF-1 carries the potential for a therapy of hearing loss. The PMN mouse has been chosen as an auditory model, since beneficial effects of peg-IGF-1 have been observed in this mouse before. The application of peg-IGF-1 starting at the second week after birth leads to a detectable otoprotective effect on the efferent cholinergic neurons and the synapses of the OHC. In addition, peg-IGF-1 attenuated the degeneration of the OHC, however, without functional benefit. This is of highest interest, as the otoprotective effect of IGF-1 on the synapse in the organ of Corti already might have beneficial effects in other forms of auditory synaptopathy, e.g., noise or age induced (53, 109). Thus, an application of peg-IGF-1 may be effective and should be tested in a clinical trial, although it must be considered, that in these cases, the afferent synapse is mainly affected.

The late start of the treatment in PMN mice might explain the missing functional effect of peg-IGF-1. The treatment could achieve a better effect by earlier application, which also means that the application must start before the onset of hearing in mice.

The described “efferent auditory neuropathy” of the MOC fibers and the OHC synapsis is an outstanding new phenotype in the PMN mouse. However, it needs to be further investigated whether during the development of the organ of Corti, the pathology of the efferent fibers and the OHC synapse leads to OHC loss in the PMN mouse. For this purpose, a postsynaptic analysis of OHC should be performed, in which the Ach receptor (110) and the calcium homeostasis (111) should be investigated.

## Data Availability Statement

The original contributions presented in the study are included in the article/supplementary material, further inquiries can be directed to the corresponding author/s.

## Ethics Statement

The animal study was reviewed and approved by Regierung von Unterfranken.

## Author Contributions

KR contributed to conception, the design of the study, and provided supervision. LB and KR wrote the manuscript. LB, JS, and JV performed audiological assessment. LB, BK, and JS organized and performed immunohistochemistry, microscopy, and statistical analysis. LB and AS initiated and created quantification of synapse entropy. LB, SJ, JV, AS, RH, and KR contributed to manuscript revision, read, and approved the submitted version. All authors contributed to the article and approved the submitted version.

## Funding

This project was funded by DFG (RA 281/-2-1). The Open Access Publication Fund of the University of Wuerzburg supported the publication.

## Conflict of Interest

The authors declare that the research was conducted in the absence of any commercial or financial relationships that could be construed as a potential conflict of interest.

## Publisher's Note

All claims expressed in this article are solely those of the authors and do not necessarily represent those of their affiliated organizations, or those of the publisher, the editors and the reviewers. Any product that may be evaluated in this article, or claim that may be made by its manufacturer, is not guaranteed or endorsed by the publisher.
